# Supplementary figures and images for: Hyperreactivity to uncertainty is a key feature of subjective cognitive impairment
Source: eLife. 2022 May 10;11:e75834. doi: 10.7554/eLife.75834 (PMC9197396; doi:10.7554/eLife.75834)

**a.**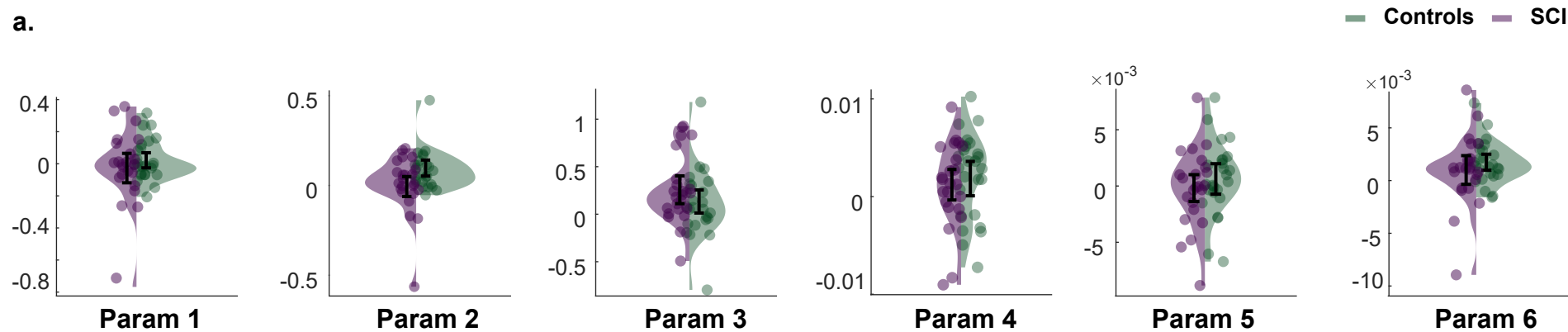**b.**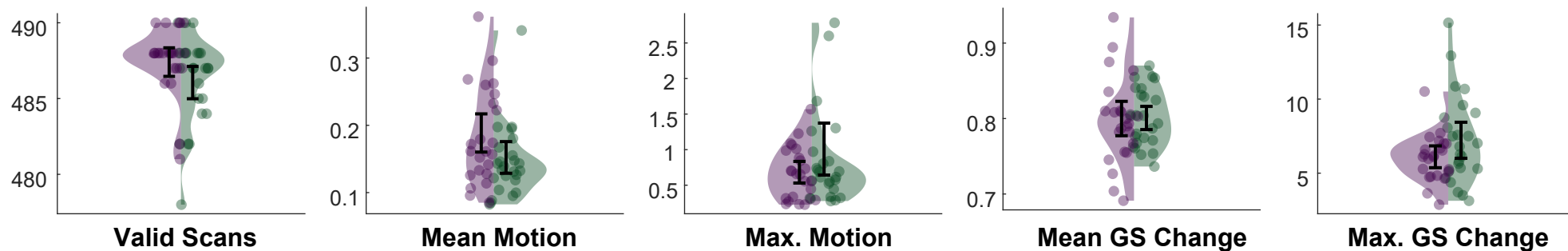

Supplement: Supplementary file 11. — (a) Six motion parameters were used during realignment procedure for rfMRI processing. These correspond to six timeseries containing three transnational and three rotational parameters over time for each subject. None of these parameters was significantly different between SCI and controls groups (all pcorr>0.61). (b) Five quality control estimates were used during preprocessing of neuroimaging data (Whitfield-Gabrieli and Nieto-Castanon, 2012). These included number of valid scans after scrubbing procedure, mean and maximum motion (extracted from the six parameters above), mean and maximum global signal change. None of these parameters was significantly different between the two groups (all pcorr>0.16 ). Based on mean motion and mean global signal changes, four potential outliers (three SCI participants and one control with values above or below Q3+1.5IQR) were identified. A second version of neuroimaging analysis was performed with these participants excluded (Supplementary file 10). There were no changes to the results or conclusions made in the paper. These findings suggest that rfMRI differences between SCI participants and controls are unlikely due to motion artifacts. Mean and max motion was calculated based on Power et al., 2012. Error bars show ± 95% CI. [file elife-75834-supp11.pdf]

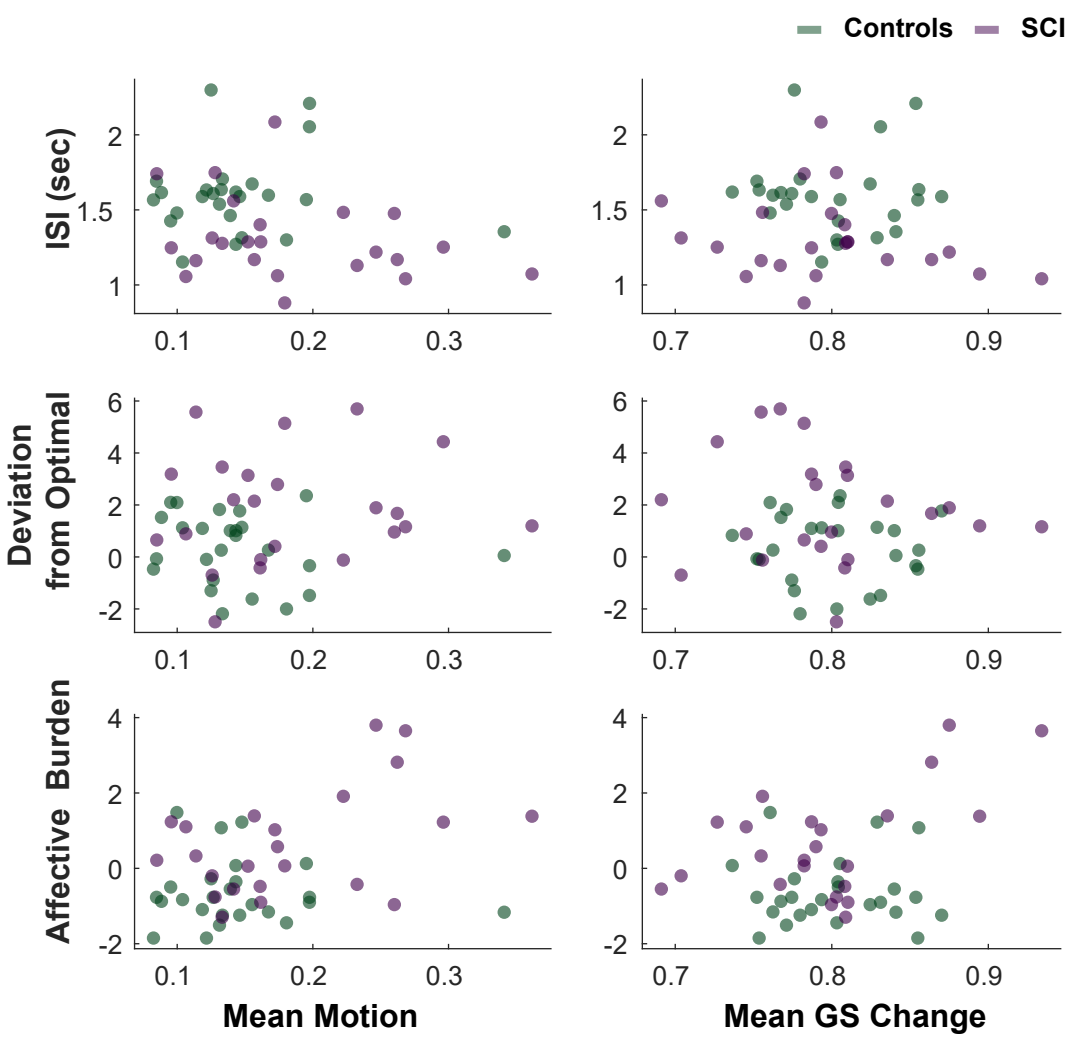

Supplement: Supplementary file 12. — Across study participants, no correlation was found between mean motion (or global signal change) and hyperreactivity to uncertainty (ISI or Deviation from optimal) or affective burden (all pcorr=1 ). Specifically, no correlation between ISI (the measure that correlates with insular-hippocampal connectivity) and these quality control measures (mean motion and mean GS change) across SCI participants (p=0.13& p=0.49 , respectively). These findings suggest that correlation between ISI and insular-hippocampal connectivity is unlikely due to motion artifacts. Correlations were controlled for age and gender. Error bars show ± 95% CI. [file elife-75834-supp12.pdf]
